# Factors Influencing the Inter‐Country Movement Intentions of Future Dentists in Ireland: A Cross‐Sectional Study

**DOI:** 10.1155/ijod/6636193

**Published:** 2026-01-22

**Authors:** Md Monirul Islam, Ave Põld, Jip Janssen, Orsolya Németh, Stefan Listl, Jostein Grytten, Francis Burke, Noel Woods

**Affiliations:** ^1^ Centre for Policy Studies, University College Cork, Cork, Ireland, ucc.ie; ^2^ Estonian Dental Association, Tallinn, Estonia; ^3^ Heidelberg Institute of Global Health, Section for Oral Health, Heidelberg University Hospital, Heidelberg University, Heidelberg, Germany, heidelberg.edu; ^4^ Department of Dentistry, Radboud University Medical Center, Nijmegen, Netherlands, radboudumc.nl; ^5^ Department of Public Dental Health, Semmelweis University, Budapest, Hungary, petho.hu; ^6^ Department of Community Dentistry, University of Oslo, Oslo, Norway, uio.no; ^7^ Department of Restorative Dentistry, Cork University Dental School and Hospital, University College Cork, Cork, Ireland, ucc.ie

**Keywords:** cross-sectional survey, future dentists, inter-country movement, ireland

## Abstract

**Introduction:**

To provide the optimum level of oral healthcare, the supply of dentists should mirror the population’s oral health care needs. However, the ongoing inter‐country movement of oral health professionals is reducing Ireland’s capacity to maintain an adequate oral health workforce. This study aimed to identify the determinants of future dentists’ intentions to engage in inter‐country movement.

**Methods:**

A cross‐sectional survey was conducted among final‐year dental students at University College Cork (UCC), Ireland. To collect the data, a Microsoft Forms based questionnaire was distributed among all students. Descriptive statistics were calculated to characterise students’ motives for pursuing a future career abroad.

**Results:**

A total of 50 out of 50 invited final‐year dental students participated in the study. The sample consisted of equal proportions of Irish nationals and international students (*n* = 25 each). Canadian students represented the predominant international cohort, accounting for 42% of all participants. Regarding immediate career plans, 8% of Irish students intended to move abroad compared with 68% of international students. The sample consisted of equal proportions of Irish nationals and international students (*n* = 25 each), with Canadian students comprising the largest subgroup at 42% of the total sample. A marked difference was observed between the two cohorts in terms of immediate career intention. Only 8% of Irish students indicated plans to work abroad, compared with 68% of international students, the majority of whom expressed a desire to return to their home country. Regarding long‐term career plans, 20% of Irish students anticipated relocating abroad, whereas only 12% of international students reported intentions to leave their country of origin. The most frequently cited push factors influencing decisions to emigrate from Ireland included more favourable economic conditions, improved lifestyle opportunities, increased access to training programmes and family‐related considerations.

**Conclusion:**

The majority of international dental students in Ireland are aiming to pursue their dental career in their home countries due to better economic conditions and living standards. These findings suggest that Irish policymakers and educational institutions might need to consider new approaches for the retention of newly graduating dentists.

## 1. Introduction

It is essential to have the required level of dental workforce to provide effective oral health care. Many high‐income countries continue to experience an increase in demand for dental service providers [1, 2]. In Ireland, the situation is more pronounced due to graduate migration, a lower number of dental graduates, and difficulties in retaining them. According to a recent report by the Irish Dental Association (IDA), a minimum of 500 additional dentists are needed in both the private sector and public service to address the escalating demand and need for dental treatment among the population. Notably, Ireland compares poorly with other European countries in terms of dentist‐to‐population ratios. According to Fernández‐Serrano et al. [4], Ireland ranks the lowest in Europe for the number of general dentists per capita, highlighting a stark disparity in workforce availability [3]. WHO data further supports this concern, reporting that Ireland has one of the lowest dentist densities among OECD countries, with approximately six dentists per ten thousand population, compared to higher ratios in countries like Germany, France and Spain [5, 6]. This is compounded by an increase in population. According to the Central Statistics Office, the estimated population was 5.3 million in 2023, with a notable increase in the older adult demographic [7]. This age group is projected to double over the next two decades. Older adults often require more extensive and specialised dental care [8]. This situation ultimately affects those seeking treatment by increasing the waiting time to receive essential oral health care, but the impact extends further. A limited dental workforce can lead to reduced access to timely preventive and restorative care, particularly in underserved or rural areas [9].

The reasons behind dentist migration are intricate, shaped by a wide array of factors that influence a dentist’s decision to migrate, which differ between countries with varying income levels [3, 5, 10–13]. Although no studies have specifically evaluated the reasons behind dentist migration from Ireland, it is assumed that factors such as better work–life balance, lower cost of living and more advanced training and career opportunities may play a role. These assumptions are informed by findings from studies on medical doctor migration, which suggest similar motivating factors [14–16]. These factors make other countries attractive destinations for dental professionals seeking to improve professional and personal lives.

To practice legally in Ireland, dentists must register with the Dental Council, which allows four different registration pathways: (a) graduating from a university in Ireland, (b) being a qualified dentist from the European Economic Area (EEA), which includes all member states of the European Union and UK, Norway, Iceland and Liechtenstein, (c) being a dentist covered by the mutual recognition agreement with Canada or New Zealand and (d) passing a Dental Council examination for graduates from universities outside the EEA and the mutual agreement acceptant countries [17].

In Ireland, students enter dental programmes through several pathways. The most common route is via the Central Applications Office (CAO) following completion of the leaving certificate or its equivalent. Additional pathways include the Leaving Certificate Access Programme (designed to support students from underrepresented backgrounds), mature student CAO entry (for applicants aged 23 or older) and entry routes for non‐EU international students [18–20]. Some non‐EU applicants may also enter through a graduate entry pathway if they have previously completed an undergraduate degree. Currently, only two institutions in Ireland, University College Cork (UCC) and Trinity College Dublin (TCD), offer dentistry programmes, collectively producing approximately 90 new graduates each year. A significant proportion of these graduates are international students, primarily from non‐EEA countries. Recently, the IDA reported that among the total student intake for 2023/24, 36% of TCD and 47% of UCC students were from non‐EEA countries [21]. These students pay tuition fees exceeding €45,000 per annum, providing significant financial support to Ireland’s underfunded dental schools [21]. A third dental school, based at the Royal College of Surgeons in Ireland, is scheduled to commence enrolling students in September 2025. Current registration figures from the Irish Dental Council do not accurately reflect the active number of practising dentists; however, workforce shortages remain evident. A 2024 IDA survey reported that 63% of dental practices that attempted to hire a dentist within the past year were unable to find a suitable candidate [21]. This highlights ongoing retention challenges, as many international graduates, despite completing their education and residing in Ireland for several years, do not remain to begin their professional careers in the country. Although international students contribute significantly to the Irish economy, there is currently no explicit policy framework prioritising their retention in the labour market—a gap that limits efforts to address workforce shortages. While national strategies, such as the National Skills Strategy 2025 and the OECD Skills Strategy Ireland highlight broader goals around skills development and lifelong learning, they do not specifically address the retention of international graduates [22, 23].

Given the lack of research on factors influencing migration among newly graduated dentists in Ireland, understanding these drivers is vital for developing strategies to improve workforce retention. This study examines the determinants shaping dental students’ intentions to migrate, providing insights into the motivations guiding career decisions abroad. Informing strategies aimed at improving retention within the national oral healthcare workforce.

## 2. Materials and Methods

### 2.1. Participants and Setting

This cross‐sectional study was conducted among all final‐year dental students studying at UCC, Ireland. Given the limited target population (a single academic cohort at one institution), a non‐randomised convenience sampling approach was used, whereby all accessible students in the cohort were invited to participate. The final sample size was 50.

### 2.2. Data Collection

A leaflet containing a Microsoft Forms survey link and a QR code was distributed to all final‐year students during a tutorial session in 2024, 2 months prior to the final examination. The survey’s purpose and administration procedures were explained during a break period, and all students were invited to complete it at their convenience. Participation was entirely voluntary, and students were assured that responses would remain anonymous and confidential. No incentives were provided. Most questionnaires were completed within the following week, although no strict deadline was imposed

### 2.3. Questionnaire

Following the initial development of the survey questionnaire, face and content validity were ensured through expert review by academics in dental education and workforce research. Feedback was used to refine item clarity, structure, and relevance. The questionnaire was informed by our concurrent systematic review on dentist migration motives [24] and by existing studies on medical migration in Ireland. While dentistry presents unique aspects—such as limited reciprocal registration agreements—relevant themes from validated tools in related fields were adapted. Although no formal statistical validation (e.g., factor analysis) was conducted, the instrument was rigorously developed through expert input and literature synthesis. The revised version of the questionnaire was piloted with five students who were not target subjects. The final questionnaire, which included 22 items, was distributed to the target group. The survey collected data on participants’ sociodemographic characteristics (e.g., age, gender, year of study, course duration, country of origin and entry pathway) and explored career intentions and migration preferences. Specifically, participants were asked about their short‐term and long‐term career plans, willingness to migrate abroad, preferred destinations and estimated duration of stay. Additional questions assessed perceptions of factors influencing inter‐country movement, including personal, professional and systemic considerations (e.g., standard of living, training opportunities and job preferences). These were presented in multiple‐choice format. Only fully completed questionnaires could be submitted.

### 2.4. Statistical Analysis

Descriptive statistics were used to summarise the data. Means and standard deviations (SD) were reported for continuous variables, and frequencies with percentages were used for categorical variables. To examine differences between Irish and non‐Irish students and between short and long term stay duration, Chi‐square tests and Fisher’s Exact tests were applied as appropriate. For the continuous variable we performed Mann–Whitney *U* test as data were not normally distributed. All analyses were conducted using SPSS version 25.0, with a significance level set at *p* < 0.05.

## 3. Results

### 3.1. Participant Characteristics

Table 1 summarises respondent characteristics. All 50 eligible students completed the questionnaire (response rate: 100%). The mean age was 25.1 years, and 66% were female. Half of the participants held Irish nationality, while the remainder were from Canada (42%), Singapore (2%), the USA (2%), Kenya (2%) and Malaysia (2%). For this study, ’Irish’ refers to individuals with Irish nationality.

**Table 1:** Characteristics of the study participants (*n* = 50).

Characteristic	Category	*n* (%) or mean ± SD
Age (years)	—	25.1 ± 3.1

Gender	Women	33 (66%)
	Men	17 (34%)

Programme duration	4 years	18 (36%)
	5 years	32 (64%)

Nationality	Ireland	25 (50%)
	Canada	21 (42%)
	Singapore	1 (2%)
	United States	1 (2%)
	Kenya	1 (2%)
	Malaysia	1 (2%)

Entry pathway	Leaving cert or equivalent CAO entry	18 (36%)
	Leaving cert access programme	2 (4%)
	Mature student CAO entry	5 (10%)
	Non‐EU international student	10 (20%)
	Non‐EU graduate entry	13 (26%)
	Other	2 (4%)

Dentistry as first‐choice programme	Yes	40 (80%)

Abbreviation: SD, standard deviation.

Among Irish students, 80% entered the Bachelor of Science Degree programme (BDS) directly through Leaving Certificate or equivalent CAO entry‐level pathways. Of these, the majority (80%) had selected dentistry as their first‐choice degree.

### 3.2. Immediate and Long‐Term Career Intentions

In Table 2, there were marked differences between Irish and international students in their immediate career intentions. While 80% of Irish students intended to work in Ireland (68% full‐time and 12% part‐time), only 12% of international students reported the same. In contrast, 68% of international students planned to work in a general dental practice outside Ireland, compared to just 4% of Irish students. This difference was statistically significant (*p* < 0.001).

**Table 2:** Career intentions after graduating as a dentist.

Immediate and long‐term plans	Nationality			
	Total (*n* = 50)	Irish (*n* = 25)	International (*n* = 25)	*p*‐Value
Immediate plans				
Taking time‐out	7 (14%)	3 (12%)	4 (16%)	0.001^a^
Working in a general dental practice in Ireland full‐time	20 (40%)	17 (68%)	3 (12%)	
Working in a general dental practice in Ireland part‐time	3 (6%)	3 (12%)	–	
Working in a general dental practice abroad (outside Ireland)	18 (36%)	1 (4%)	17 (68%)	
Undertaking foundation/vocational training abroad/outside their home country	2 (4%)	1 (4%)	1 (4%)	
Long‐term plans (multiple answers possible)				
Working in a general dental practice in Ireland full‐time	31 (62%)	14 (56%)	17 (68%)	0.404^a^
Working in a general dental practice in Ireland part‐time	5 (10%)	4 (16%)	1 (4%)	
Working as a specialist dentist	16 (32%)	8 (32%)	8 (32%)	
Working in facial aesthetics part‐time	10 (20%)	7 (28%)	3 (12%)	
Working in facial aesthetics full‐time	1 (2%)	–	1 (4%)	
Working abroad/outside their home country	8 (16%)	5 (20%)	3 (12%)	
In a teaching position	5 (10%)	4 (16%)	1 (4%)	

^a^Chi‐square test.

In terms of long‐term intentions, both Irish and international students reported similar preferences. In both groups, 72% planned to work in a general dental practice in Ireland, either full‐time or part‐time, and 32% expressed interest in specialist careers. Some students also indicated preferences for facial aesthetics and teaching roles. No statistically significant differences were observed between groups.

It should be noted that participants could select multiple options, which may limit interpretation of a single preferred career trajectory or primary motive.

### 3.3. Factors Influencing Inter‐Country Movement Intentions

We explored patterns in migration intentions among future dentists, with respect to duration of stay and nationality.

#### 3.3.1. Migration Intentions by Planned Duration of Stay

As detailed in Table 3, twenty‐two respondents expressed an intention to move abroad immediately after graduation. Of these, the majority (*n* = 17) planned to relocate for the long term (>5 years), while a smaller group (*n* = 5) indicated short‐term intentions (≤5 years).

**Table 3:** Factors influencing inter‐country movement intention according to time frame (based on immediate plans after graduation).

Category	Duration of stay		
	Short‐term (≤5 years) (*n* = 5)	Long‐term (>5 years) (*n* = 17)	*p*‐Value
Gender			
Women	2 (40%)	11 (64.7%)	0.609^a^
Men	3 (60%)	6 (35.3%)	
Age (years)	23.4 ± 1.5	26.3 ± 2.3	0.013^b^
Nationality			
Irish	2 (40%)	1 (5.9%)	0.023^a^
Canada	2 (40%)	16 (94.1%)	
USA	1 (20%)	–	
Destination country			
Canada	2 (40%)	16 (94.1%)	0.023^a^
UK	2 (40%)	1 (5.9%)	
Australia	1 (20%)	–	
Motivating factors			
Access to foundation/vocational training	1 (20%)	5 (29.4%)	1.000^a^
Better dental remuneration	1 (20%)	4 (23.5%)	1.000^a^
Better working conditions	2 (40%)	4 (23.5%)	0.630^a^
Better economic conditions	1 (20%)	5 (29.4%)	1.000^a^
Better lifestyle	3 (60%)	5 (29.4%)	0.240^a^
Advanced training opportunities	1 (20%)	5 (29.4%)	1.000^a^
Family reasons	–	6 (35.3%)	0.029^a^

*Note*: Table includes respondents who indicated plans to move abroad and provided their intended duration of stay.

^a^Fisher’s Exact test.

^b^Mann–Whitney *U* test.

Canada was the most frequently selected destination among those planning a long‐term stay (94.1%), whereas short‐term plans were more evenly distributed, with Canada and the UK each selected by 40% of respondents.

Nationality was significantly associated with duration of stay (*p* = 0.023). Canadian nationals were more likely to plan long‐term relocation (94.1%), whereas Irish nationals were predominantly associated with short‐term plans.

In terms of motivating factors, respondents planning to stay short term most frequently cited a better lifestyle (60%) as the primary reason for moving abroad. Among those intending long‐term relocation, the most commonly reported factors were family reasons (35.3%), better economic conditions (29.4%), advanced training opportunities (29.4%) and a better lifestyle (29.4%). Family reasons were the only factor significantly associated with duration of stay (*p* = 0.029), suggesting that long‐term movers are more strongly influenced by familial ties than those planning short‐term stays.

#### 3.3.2. Migration Intentions by Nationality

As shown in Table 4, three Irish nationals and nineteen international students reported plans to work abroad after graduation. Canada was the most frequently selected destination overall, chosen by 89.5% of international students compared with 33.3% of Irish students. In contrast, Irish respondents were more likely to select the United Kingdom, with 66.7% indicating it as their preferred destination.

**Table 4:** Factors influencing inter‐country movement intentions according to nationality (immediately after graduation).

Category	Nationality		
	Irish (*n* = 3)	International (*n* = 19)	*p*‐Value
Gender			
Women	2 (66.7%)	12 (63.2%)	1.00^a^
Men	1 (33.3%)	7 (36.8%)	
Age (years)	26.3 ± 2.1	25.6 ± 3.2	0.009^b^
Planning to work abroad			
Long‐term (>5 years)	2 (66.7%)	16 (84.2%)	0.470^a^
Short term (≤5 years)	1 (33.3%)	3 (15.8%)	
Purpose			
Working general dental practice	2 (66.7%)	18 (94.7%)	0.259^a^
Undertaking foundation/vocational training	1 (33.3%)	1 (5.3%)	
Destination Country			
Canada	1 (33.3%)	17 (89.5%)	0.072^a^
UK	2 (66.7%)	2 (10.5%)	
Motivating factors toward destination selection			
Access to foundation/vocational training	1 (33.3%)	6 (31.6%)	1.00^a^
Better dental remuneration	1 (33.3%)	3 (15.8%)	0.60^a^
Better working conditions	1 (33.3%)	5 (26.3%)	1.00^a^
Better economic conditions	–	6 (31.6%)	0.21^a^
Better lifestyle	2 (66.7%)	7 (36.8%)	0.57^a^
Advanced training opportunities	1 (33.3%)	5 (26.3%)	1.00^a^
Family reasons	—	7 (36.8%)	0.12^a^

*Note*: Based on respondents (*n* = 22) intending to migrate abroad short‐term (≤5 years) or long‐term (>5 years), as outlined in Table 3.

^a^Fisher’s Exact test.

^b^Mann–Whitney *U* test.

Most respondents, regardless of nationality, planned to work in general dental practice. Specifically, 94.7% of international students and 66.7% of Irish students stated this as their primary purpose for working abroad. While only one respondent from each group reported an interest in undertaking foundation or vocational training, and this difference was not statistically significant. No significant differences were observed between Irish and international students in terms of gender distribution or age.

Regarding factors influencing destination choice, both groups reported similar motivations. A better lifestyle was cited by 66.7% of Irish respondents and 36.8% of international students. Other commonly mentioned factors included access to foundation or vocational training, with no significant differences observed between groups. Family reasons were reported exclusively by international students (36.8%), although this difference was not statistically significant.

Table 5 presents the distribution of motivating factors among respondents who selected Canada (*n* = 17) as their preferred long‐term destination (>5 years). The most frequently cited motivating factors were better economic conditions (52.9%), a better lifestyle (47.1%) and family reasons (41.2%). Other factors included access to foundation or vocational training (41.2%), better dental remuneration (29.4%), better working conditions (23.5%) and opportunities for greater capacity for advanced training (17.6%).

**Table 5:** Disaggregated motivating factors for respondents (*n* = 17) who selected canada as a preferred long‐term destination ( >5 years).

Motivating factor	*n* (%)
Better economic conditions	9 (52.9%)
Better lifestyle	8 (47.1%)
Family reasons	7 (41.2%)
Access to foundation/vocational training	7 (41.2%)
Better dental remuneration	5 (29.4%)
Better working conditions	4 (23.5%)
Greater capacity for advanced training	3 (17.6%)

*Note:* Multiple responses were allowed.

### 3.4. Motivating Factors for Migration

Respondents considering or committed to moving abroad most frequently cited a more favourable lifestyle as the primary push factor followed by family presence in the destination country and better working conditions. Other commonly reported considerations included opportunities for advanced training, improved economic conditions, higher dental remuneration and access to foundation or vocational training.

Overall, migration intentions appeared to be driven by a combination of lifestyle, family and professional factors, with lifestyle and family ties emerging as the most prominent.

## 4. Discussion

The shortage of dental professionals is an emerging challenge both in Ireland and globally, raising concerns about workforce sustainability and equitable access to care. In Ireland, workforce shortages have been reported across public and private sectors, contributing to delays in care delivery and reduced service capacity. Our findings indicate that workforce shortages may be further exacerbated by the outflow of newly qualified dentists. Among our respondents, a significant proportion of both Irish and international dental students expressed intentions to migrate abroad after graduation, with many planning long‐term relocation. These trends echo findings from studies on Irish medical graduates, where 50%–80% reported an intention to migrate [14, 25, 26]. The high proportion of dental students considering long‐term emigration, particularly to countries like Canada, raises concerns about the sustainability of the domestic dental workforce. The implications of these findings are considerable not only due to the substantial public and personal investment required to train dental professionals, but also in terms of recruitment challenges, service disruptions, and the increasing burden placed on the remaining workforce. Although dental migration may not be the sole cause of the dentist shortage in Ireland, our findings suggest it is a significant contributing factor that warrants focused attention in workforce planning and policy development.

In this research, 50% of respondents were international students (*n* = 25), the majority of whom were Canadian citizens. This is likely due to the recognition of dental qualifications between Ireland and Canada. Dental degrees earned in Ireland are recognised by the National Dental Examining Board of Canada (NDEB), which enables Irish graduates to pursue licensure through the accredited pathway. Similarly, Canadian dental degrees are recognised by the Dental Council of Ireland, allowing Canadian graduates to register and practise without additional assessments. This two‐way recognition greatly supports professional mobility, which helps to explain the strong preference for Canada that exists among Irish and international students [27]. Although there are no estimates on the number of Irish students studying dentistry in Canada, it is highly unlikely the number is significant [28].

In our analysis, 68% of Irish students reported an intention to work full‐time in Ireland following graduation, while in case of immediate plan after graduation expressed a desire to work full‐time in Ireland, whereas an equal percentage of international students indicated an intention to work outside Ireland or return to their home country. Immediate settlement might be a reason in case of Irish student for immediate stay decision in Ireland. In contrast, in our sample, a substantial proportion of international students were Canadian. These students may perceive that returning home offers better conditions for repaying educational debt, whether due to potential remuneration, greater familiarity with the practice environment or broader professional and personal considerations. While these early intentions are understandable, it is also important to note that it may be difficult for students to plan definitively before entering professional practice. Interestingly, in terms of long‐term plans, there was a noticeable shift. Where, more Irish students expressed a desire to migrate abroad compared to international students. This finding is concerning for the future of the Irish dental workforce. However, responses may have been influenced by the structure of the questionnaire, which allowed for multiple answers, and by students limited professional experience at this early stage.

A previous study in New Zealand [3] found that 64% of international students intended to remain in the host country after graduation—a figure much higher than that observed among international students in Ireland. Furthermore, retrospective data from the same study indicated that 22% of international graduates were actually working in the host country post‐graduation. These findings suggest that migration intentions may evolve over time. Future follow‐up studies could help clarify trends in post‐graduation movement and the transition of international students into the workforce.

In our analysis, respondents selecting Canada as their preferred long‐term destination (*n* = 17), the main motivating factors were better economic conditions (52.9%), improved lifestyle (47.1%) and family reasons (41.2%). Other notable factors included access to foundation/vocational training (41.2%), better remuneration (29.4%), working conditions (23.5%) and advanced training opportunities (17.6%). These findings align with previous literature. For example, a study of Romanian dental students also identified the prospect of higher income abroad as a key motivator for migration [5]. Although Ireland offers competitive dentist salaries, the higher cost of living and the financial demands of establishing a dental practice may offset these earnings compared to some regions in Canada [29–31]. Given that a large proportion of international students in this study were Canadian, this comparison is particularly relevant.

In terms of access to foundational and vocational training, 29.4% of those with the motivation to move abroad mentioned it as a factor. In the context of dentistry, foundational training generally refers to a structured 1‐year postgraduate programme for newly qualified dentists, designed to provide supervised clinical experience within a general dental practice setting In the context of dentistry, foundational training generally refers to a structured 1‐year postgraduate programme for newly qualified dentists, designed to provide supervised clinical experience within a general dental practice setting [32]. This stage is considered a critical step for dentists seeking to practice independently [33]. Currently, a formal foundation training programme is not available in Ireland. The absence of such structured training opportunities may serve as a significant driver for Irish dental graduates seeking to relocate abroad [29]. As a result, this lack of foundation training opportunities plays role in motivating Irish dental graduates to move abroad. It is noteworthy that 56% of Irish graduates intend working full‐time in the long‐term, Table 2. Our findings also indicate emerging trends, such as increasing interest in facial aesthetic procedures among dental students, which may contribute to reduced participation in traditional full‐time dental practice in Ireland.

A substantial proportion of students who desire to move from Ireland cite more favourable working conditions and lifestyles as motivation factors. Although Ireland’s economic development is progressing [34], Ireland had the second highest cost of living in the EU in 2023 according to Eurostat [35], which is 46% above the EU average, particularly driven by housing and health costs. Similarly, data from Numbeo [36 ] regularly shows that cities like Dublin have a higher cost of living than many Canadian cities, such as Toronto or Vancouver, especially when considering rent and healthcare costs. It is reasonable to assume that countries with a lower cost of living may enable dentists to attain a comparatively higher standard of living, even when earning an equivalent or lower salary. Additionally, staffing crises in dentistry forces practising dentists to work under substantial pressure [37]. This can result in long working hours, more stress and a higher risk of burnout [38, 39]. In spite of this only 20% of graduates who are Irish intend working abroad in the long term.

As most international dental students in Ireland are from Canada, the current retention plans may be too narrowly aligned with their priorities. To effectively address workforce shortages, future strategies should focus on broadening the diversity of international student recruitment and identifying what would motivate Canadian graduates to remain in Ireland. Without understanding and tackling these challenges, an effort to reverse the current pattern of graduate emigration will remain limited in impact.

### 4.1. Strengths and Limitations

This study has several strengths. First, the response rate was 100%, which decreases the likelihood of a response bias due to participant non‐response. While item non‐response was minimal, missing data was carefully addressed to ensure the reliability of the analysis. Second, this is the first study among future dentists that compares the motives of inter‐country movement between the local and international groups.

This study has several limitations. First, the findings only represent a small number of participants from one dental school out of two (UCC and TCD) and may not be widely applicable. Although both UCC and TCD follow national curricula, differences in institutional settings, clinical training or local patient populations may influence students’ experiences and perceptions. Second, only final‐year dental students were included, which could affect the generalizability of the findings. Also, the findings may differ for students in other years, and there is a possibility that their motivation may change following graduation. Conducting surveys of final‐year students over multiple consecutive years could help to assess the consistency of the migration motivation. Third, the questionnaire design grouped into multiple related push factors together, such as economic factors and lifestyle considerations. While this approach streamlined the analysis, it may have obscured important distinctions between specific motivations. Future studies with larger sample sizes should consider analysing these elements separately to provide a more detailed understanding of movement intentions. Fourth, due to resource limitations, in this study Irish students studying in Canada were not included. Their motivations for returning to Ireland could have provided valuable insights for this research.

## 5. Conclusions

Ireland’s dental workforce is facing significant challenges. This study highlights many Irish and international dental students intend to migrate after graduation and this is driven by economic factors, lifestyle preferences and the availability of structured postgraduate training abroad. To address this issue, workforce planning must prioritise improving retention. Without targeted strategies, Ireland risks continued shortages and reduced access to dental care.

## Author Contributions

Conceptualisation, supervision: Noel Woods and Stefan Listl. Methodology, software, formal analysis, writing – original draft preparation: Md Monirul Islam. Validation: Ave Põld, Jip Janssen and Orsolya Németh. Investigation: Md Monirul Islam and Francis Burke. Resources, project administration, funding acquisition: Noel Woods. Data curation: Md Monirul Islam and Ave Põld. Writing – review and editing: Orsolya Németh, Francis Burke and Jostein Grytten.

## Funding

This study is a part of the PRUDENT project and has received funding from the European Union’s Horizon Europe research and innovation programme (Grant 101094366): https://cordis.europa.eu/project/id/101094366.

## Disclosure

All the authors have read and agreed to the published version of the manuscript.

## Ethics Statement

Ethical approval for this study was received from Social Research Ethics Committee (SREC) of the University College Cork (Reference Number Log 2023‐216 on 20 November 2023).

## Conflicts of Interest

The authors declare no conflicts of interest.

## Data Availability

The dataset without identification, which was used during the current study, is available from the corresponding author upon reasonable request.
